# Modulation of Gonadotropin-Releasing Hormone Neuron Activity and Secretion in Mice by Non-peptide Neurotransmitters, Gasotransmitters, and Gliotransmitters

**DOI:** 10.3389/fendo.2019.00329

**Published:** 2019-05-22

**Authors:** Daniel J. Spergel

**Affiliations:** Department of Neurosurgery, Yale University School of Medicine, New Haven, CT, United States

**Keywords:** GnRH neurons, neurotransmitters, gasotransmitters, gliotransmitters, signaling pathways, electrophysiology, calcium, GnRH secretion

## Abstract

Gonadotropin-releasing hormone (GnRH) neuron activity and GnRH secretion are essential for fertility in mammals. Here, I review findings from mouse studies on the direct modulation of GnRH neuron activity and GnRH secretion by non-peptide neurotransmitters (GABA, glutamate, dopamine, serotonin, norepinephrine, epinephrine, histamine, ATP, adenosine, and acetylcholine), gasotransmitters (nitric oxide and carbon monoxide), and gliotransmitters (prostaglandin E2 and possibly GABA, glutamate, and ATP). These neurotransmitters, gasotransmitters, and gliotransmitters have been shown to directly modulate activity and/or GnRH secretion in GnRH neurons *in vivo* or *ex vivo* (brain slices), from postnatal through adult mice, or in embryonic or immortalized mouse GnRH neurons. However, except for GABA, nitric oxide, and prostaglandin E2, which appear to be essential for normal GnRH neuron activity, GnRH secretion, and fertility in males and/or females, the biological significance of their direct modulation of GnRH neuron activity and/or GnRH secretion in the central regulation of reproduction remains largely unknown and requires further exploration.

## Introduction

Gonadotropin-releasing hormone (GnRH) neurons, whose cell bodies (in rodents) reside mainly in the preoptic area (POA) of the hypothalamus as well as in the medial septum (MS) and diagonal band of Broca (DBB), provide the final output in the central regulation of mammalian fertility: pulsatile and, in females, surge GnRH secretion from axon terminals in the median eminence (ME). This is despite the fact that subpopulations of kisspeptin (KP) neurons in the arcuate nucleus (ARC) and rostral periventricular area of the third ventricle (RP3V), which innervate GnRH neurons, appear to be the “GnRH pulse generator” and “GnRH surge generator,” respectively, and together with KP neurons in the posterodorsal medial amygdala (MePD) integrate endocrine, metabolic, and environmental (including circadian, stress, pheromonal, and sexual behavior-related) signals in the hypothalamic-pituitary-gonadal axis (1–11). These signals are conveyed to GnRH neurons by KP neurons, other neurons, and glial cells that release one or more peptide neurotransmitters (including KP), non-peptide neurotransmitters (also called conventional, classical, or small-molecule neurotransmitters), gasotransmitters, and/or gliotransmitters onto GnRH neuron cell bodies, dendrites, or “dendrons” to modulate GnRH neuron activity (considered here to be changes in membrane potential, action potential firing, and/or cytoplasmic, free Ca^2+^ concentration ([Ca^2+^]_i_)) and GnRH secretion (12–28). The dendrons are processes (blended dendrites and axons), found so far only in GnRH neurons, that receive synaptic input like dendrites but also conduct action potentials like axons, and that project from GnRH cell bodies to the ME where they split into short axon terminals to enable GnRH secretion into the portal vasculature (21, 29). GnRH neurons receive their highest density of synaptic innervation along their proximal dendrons (up to ~500 μm from the cell body), which contain the action potential initiation site (30, 31). At their distal dendrons (~500 μm from the cell body), GnRH neurons appear to be interconnected through dendro-dendritic bundling and associated shared inputs from ARC KP neurons and possibly other neurons and glial cells, which may provide a mechanism for the synchronization of GnRH neuron activity necessary for pulsatile GnRH secretion, as well as an additional mechanism for its direct modulation (4, 32–36).

GnRH secreted from GnRH neuron axon terminals in the ME into the hypothalamo-hypophyseal portal circulation binds to GnRH receptors on pituitary gonadotrophs and stimulates the synthesis and secretion into the systemic circulation of luteinizing hormone (LH) and follicle-stimulating hormone (FSH). LH and FSH are required for gametogenesis and gonadal biosynthesis in both sexes, for secretion of estradiol (E, a.k.a. E2) and progesterone (P), as well as for ovulation, in females, and for secretion of testosterone (T) in males (37). E, P, and T feed back onto the brain to inhibit GnRH secretion (via inhibition of ARC KP or other neurons), and onto the anterior pituitary to inhibit LH/FSH secretion, except in the afternoon on the day of proestrus in females, when E and P stimulate a surge of GnRH secretion [via activation of RP3V KP neurons (6)]. The switch from E negative feedback to E positive feedback at proestrus is triggered by circadian input from vasopressin neurons in the suprachiasmatic nucleus (SCN) to RP3V KP neurons and GnRH neurons, and by increased levels of E. This switch, combined with E-mediated *de novo* synthesis of P within the hypothalamus, initiates the GnRH surge, which in turn triggers the LH surge that results in ovulation (6). The GnRH/LH surge is associated with increased activation of RP3V KP neurons, which (unlike ARC KP neurons) project to the soma and proximal dendrites of a subpopulation of GnRH neurons, along with increased GnRH neuron somatic and dendritic spine density, and critically depends on KP receptor signaling in GnRH neurons (1, 3, 33, 38). This suggests that an increase in excitatory synaptic input from RP3V KP neurons to a subpopulation of GnRH neurons is required for the GnRH/LH surge; however, as discussed below, the GnRH/LH surge may also depend on excitatory input from other cells (28, 39).

Owing to the relative ease with which mouse GnRH neurons can be genetically manipulated, electrophysiologically assayed, and imaged, mice represent the most tractable system for studying mammalian GnRH neurons. I recently reviewed the direct modulation of GnRH neuron activity and GnRH secretion in mice by neuropeptides, with direct modulation considered to be modulation that persists in the presence of the Na^+^ channel blocker tetrodotoxin (TTX) and amino acid transmitter receptor antagonists (40). Here, I review the direct modulation by non-peptide neurotransmitters, gasotransmitters, and gliotransmitters, including their cognate receptors and signaling mechanisms, of GnRH neuron activity and GnRH secretion in mice. The review is based on data obtained from green fluorescent protein (GFP)- or genetically encoded Ca^2+^ indicator (GECI)-expressing GnRH neurons *in vivo* or *ex vivo* (brain slices), from postnatal through adult mice, when data are available, or from alternatives such as embryonic mouse GnRH neurons or immortalized cell lines of mouse GnRH neurons (19, 25, 41–49).

## Modulation of GnRH Neuron Activity and Secretion by Non-Peptide Neurotransmitters

Non-peptide neurotransmitters are defined here as endogenous chemicals (other than neuropeptides) that transmit signals across a synapse from presynaptic neurons, where they are released from secretory vesicles via Ca^2+^-dependent exocytosis, to postsynaptic neurons (e.g., GnRH neurons), where they bind to and activate ionotropic and/or metabotropic receptors. Activation of ionotropic receptors results in the opening of ion channels through which one or more types of ions such as Na^+^, K^+^, Cl^−^, and Ca^2+^ flow, whereas activation of metabotropic receptors results in second messenger activation or inhibition of ion channels via signal transduction mechanisms that often involve G proteins. This, in turn, results in a change, or modulation, of the activity (membrane potential and/or [Ca^2+^]_i_) and output (neurotransmitter or neuropeptide release) of the postsynaptic neurons.

### Amino Acids

#### γ-aminobutyric Acid (GABA)

GABA released from non-GnRH neurons, and potentially from a subpopulation of GnRH neurons (50), binds to GABA_A_ receptors (GABA_A_Rs), which are GABA-gated Cl^−^ channels, and to GABA_B_ receptors (GABA_B_Rs), which are G protein-coupled receptors (GPCRs) linked to K^+^ channels, in GnRH neurons. This results in modulation of GnRH neuron activity and GnRH/LH secretion, with net GABA effects determined by the ratio of GABA_A_R and GABA_B_R-mediated effects (19, 26, 50–55).

Activation of GABA_A_Rs in GnRH neurons of pubertal and adult mice has complex effects on GnRH neurons but mainly depolarizes and excites them (i.e., increases their action potential firing frequency). Exogenous GABA, the GABA_A_R agonist muscimol, and the GABA_A_R antagonist bicuculline increase, decrease, or have mixed effects on action potential firing frequency that may depend on GnRH neuron resting membrane potential and/or intracellular Cl^−^ concentration, which may in turn depend on ongoing GABA_A_R signaling by endogenous GABA (19, 56–58). However, the GABA_A_R antagonist picrotoxin, which prevents activation of GABA_A_Rs by endogenous GABA, consistently suppresses GnRH neuron firing (19, 59), and GABA increases [Ca^2+^]_i_ in GnRH neurons (60, 61), suggesting that GABA predominantly depolarizes, or depolarizes and excites, GnRH neurons. GABA increases [Ca^2+^]_i_ by first opening GABA_A_R channels, which results in depolarization followed by Ca^2+^ entry through voltage-gated Ca^2+^ channels (61, 62). Additional evidence for GABAergic excitation of adult GnRH neurons via GABA_A_R activation is that most adult GnRH neurons express the Cl^−^ accumulator transporter NKCC1 and anion exchanger AE2, which contribute to depolarization after GABA_A_R activation, but not the primary Cl^−^ extruder transporter KCC2, which contributes to hyperpolarization after GABA_A_R activation (63, 64).

Adult GnRH neurons appear to express α1, α2, α3, α5, β1, β2, β3, γ1, γ2, δ, ε, and ρ1 GABA_A_R subunits, with some differences between males and females (65–70). Spontaneous release of endogenous GABA onto GnRH neurons evokes phasic postsynaptic currents mediated by γ2 subunit-containing GABA_A_Rs as well as tonic extrasynaptic currents mediated by γ2 subunit- and δ subunit-containing GABA_A_Rs (52, 71). The amplitude and frequency of GABAergic postsynaptic currents (PSCs) are reduced by 70 and 77%, respectively, and the response to exogenous GABA is reduced by 90%, in GnRH neurons of mice in which the γ2 subunit is knocked out specifically in GnRH neurons (71). Taken together with the finding that the δ subunit appears to be expressed in only 44% of GnRH neurons (52), this indicates that the γ2 subunit is critical for normal GABA_A_R function in GnRH neurons. Although male and female GnRH neuron γ2 knockout mice exhibit normal fecundity, estrous cycles, and puberty onset, suggesting that GABA_A_R-mediated neurotransmission at the GnRH neuron is not essential for GnRH secretion or fertility, it is also possible that other GABA_A_R subunits, other transmitters, or an intrinsic conductance may have compensated for the loss of the γ2 subunit in those mice (71). A 4,5,6,7-tetrahydroisoxazolo[5,4-c]pyridin-3-ol (THIP)-sensitive tonic GABAergic current modulates GnRH neuron activity, suggesting a role for δ subunit-containing GABA_A_Rs in GnRH neurons, which confer neurosteroid sensitivity, as well as a potential role for stress-derived neurosteroid modulation in the regulation of fertility [(52), reviewed by (72)]. GABA_A_Rs are expressed in the cell bodies, dendrites, and dendrons of GnRH neurons (21, 44, 62).

GABA modulates GnRH neuron activity through GABA_B_Rs in addition to GABA_A_Rs. Exogenous GABA (in the presence of the GABA_A_R antagonist picrotoxin) and the GABA_B_R agonist baclofen directly hyperpolarize GnRH neurons, by inducing a Ba^2+^-sensitive outward K^+^ current, and decrease GnRH neuron firing (51, 53, 54). Endogenous GABA inhibits GnRH neurons via GABA_B_Rs following excitation mediated by GABA_A_Rs or KP receptors (73). GABA_B_Rs in GnRH neurons are composed of GABA_B1_ and GABA_B2_ subunits as in other neurons and are expressed in 22% of GnRH neurons in male mice and 70% of GnRH neurons in female mice, with expression remaining relatively constant across the estrous cycle (51, 53, 69). Female GABA_B1_ knockout mice exhibit disrupted estrous cyclicity and reduced fertility, as well as increased expression levels of genes including Gad1 (which codes for glutamate decarboxylase, the enzyme that catalyzes the production of GABA from glutamate), Kiss1 (which codes for KP), and Gnrh1 (which codes for GnRH) that may affect the sexual differentiation of the brain and the proper wiring of the GnRH and KP systems (74, 75). However, the effects on GnRH neuron activity, GnRH secretion, or fertility of knocking out GABA_B1_ and/or GABA_B2_ subunits specifically in GnRH neurons have yet to be reported.

GABAergic wiring and transmission onto GnRH neurons appear to be important for fertility. GnRH neuron cell bodies and dendrites receive inputs from GABAergic neurons in the ARC and RP3V (26, 35, 76). GnRH neurons in the POA of prenatally androgenized (PNA) female mice receive an increased number of appositions and increased GABAergic transmission from GABAergic neurons in the ARC (35, 77). This results in increased GABAergic PSC amplitude and frequency, increased firing, persistently elevated GnRH/LH release frequency, and reduced progesterone (P) negative feedback prior to androgen (T) excess and reproductive impairments (including disrupted reproductive cycles) that mimic those of polycystic ovary syndrome (PCOS), the most common endocrinopathy in women of reproductive age and the leading cause of female infertility (35, 59, 77–80). This suggests that normal GABAergic wiring and transmission onto GnRH neurons is essential for normal GnRH neuron firing, GnRH/LH secretion, and reproductive function in females, and that increased GABAergic wiring and transmission onto GnRH neurons may play an important role in PCOS. However, it should be emphasized that, while PCOS is associated with increased cerebrospinal fluid concentrations of GABA, PCOS has a complex etiology which includes genetic, environmental, and metabolic factors along with neuroendocrine factors (78, 81).

GABA also appears to be important for activating the GnRH neurons that trigger ovulation. Low-frequency (2 Hz) *in vitro* optogenetic stimulation of GABAergic neurons in the RP3V, which co-express KP (82), generates an immediate and transient GABA_A_R-mediated increase in GnRH neuron firing, whereas higher frequencies (10 Hz) recruit the long-lasting activation observed following RP3V KP neuron stimulation (26). However, 2-Hz activation of RP3V GABAergic neurons *in vivo* does not alter LH secretion, whereas 10-Hz stimulation evokes a sustained large increase in LH secretion identical to RP3V KP neuron activation, suggesting that KP, rather than GABA, is the functionally dominant co-transmitter at the time of ovulation (26). Yet, KP may exert some of its effects on GnRH neurons via GABA. GABAergic PSC (and miniature PSC; mPSC) frequency in GnRH neurons increases (along with GnRH neuron firing rate and burst frequency) during the afternoon of proestrus in normally cycling female mice (83, 84), and KP increases the amplitude and frequency of GABAergic PSCs in GnRH neurons from ovariectomized mice treated with E (85), whose serum level rises during the afternoon of proestrus. E-dependent GABAergic transmission may be important for reproduction in females, as female mice lacking estrogen receptor α (ERα, also called ESR1) in their GABAergic neurons are infertile and have abnormal estrous cycles and abolished E positive feedback responsible for the proestrus GnRH/LH surge required for ovulation (39). In addition to acting indirectly on GnRH neurons via ERα expressed in GABAergic neurons (60), E acts directly on GnRH neurons via the estrogen receptor β (ERβ, also called ESR2)/Akt/neuronal nitric oxide synthase (nNOS) pathway to generate nitric oxide synthase (NOS) that retrogradely accelerates GABA and glutamate release from presynaptic terminals contacting GnRH neurons, which increases mPSC frequency and firing rate and thus may also contribute to the proestrus GnRH/LH surge (86).

GABA may also mediate the effects of leptin, an important regulator of food intake and energy expenditure, on GnRH neurons, which do not express leptin receptors. Mice lacking leptin receptors in their GABAergic neurons exhibit delayed puberty onset and decreased fertility in both sexes, as well as a suppressed proestrus GnRH/LH surge (87). Leptin-responsive GABAergic neurons in the ARC project to the POA (88), but it has not yet been reported whether they project to GnRH neurons in the POA and affect GnRH neuron activity or secretion.

#### Glutamate (Glu)

Glu increases GnRH neuron activity via α-amino-3-hydroxy-5-methyl-4-isoxazolepropionic acid (AMPA)/kainate (KA), N-methyl-D-aspartate (NMDA), and metabotropic glutamate (mGlu) receptors (AMPARs, NMDARs, and mGluRs) expressed in the cell bodies, dendrites, and dendrons of GnRH neurons (21, 44, 62, 89, 90). Glu may be released onto GnRH neurons from KP neurons in the ARC as well as from other neurons, possibly including GnRH neurons, since they express vesicular glutamate transporter 2 [vGluT2, (55, 70, 76, 82, 90, 91)].

AMPA increases GnRH neuron firing (19, 54), as well as Ca^2+^ entry through AMPARs, which are non-selective cation channels (61). GnRH neurons appear to express GluA1, GluA2, GluA3, and GluA4 AMPAR subunits (67, 69, 70).

NMDA increases [Ca^2+^]_i_ in only about 20% of GnRH neurons (61), probably via Ca^2+^ entry through NMDAR channels, which are non-selective cation channels with higher Ca^2+^ permeability than AMPARs, as well as via membrane depolarization followed by Ca^2+^ entry through L-type Ca^2+^ channels, and subsequently via Ca^2+^-induced Ca^2+^ release involving internal Ca^2+^ stores and IP_3_ receptors. GnRH neurons appear to express GluN1, GluN2B, GluN2D, and GluN3A NMDAR subunits (44, 67, 69, 70, 92).

The mGluR1/mGluR5 agonist (S)-3,5-dihydroxyphenylglycine (DHPG) increases firing in a subpopulation of GnRH neurons (45% in whole-cell recordings and 23% in cell-attached recordings) in the MS/DBB (90) but not in the POA (93). The DHPG-induced increase in GnRH neuron firing in the MS/DBB may occur via G_q_ protein stimulation of phospholipase C (PLC) resulting in the generation of inositol 1,4,5-trisphosphate (IP3) and diacylglycerol (DAG), followed by DAG activation of transient receptor potential-canonical (TRPC) channels, which are non-selective cation channels. GnRH neurons appear to express mGluR1, mGluR5, and mGluR8 mGluRs (67, 70).

Glu may play an important role in the modulation of GnRH neuron activity and secretion required for fertility. Both the decrease in GnRH neuron firing during E negative feedback and the increase in GnRH neuron firing during E positive feedback depend on Glu neurotransmission (15). Moreover, female mice lacking ERα in their glutamatergic neurons, which include most ARC KP neurons but only a small percentage of RP3V KP neurons, exhibit advanced puberty onset and abnormal negative feedback, are infertile, and have abolished E positive feedback responsible for the proestrus GnRH/LH surge (39). Glu neurotransmission onto GnRH neurons, at least through GluA2-containing AMPARs or all NMDARs, does not appear to be essential for normal GnRH or fertility, as shown using mice lacking GluA2-containing AMPARs or all NMDARs in GnRH neurons and other mainly limbic system neurons in hypothalamic and septal areas (94). However, other neurotransmitter receptors may have compensated during development for the lack of GluA2-containing AMPARs or all NMDARs in GnRH neurons and other mainly limbic system neurons in those mice.

### Monoamines

#### Dopamine (DA)

DA exerts complex pre- and postsynaptic actions on GnRH neurons. Exogenous DA decreases firing via direct postsynaptic actions, as well as indirectly via RP3V-evoked GABAergic and glutamatergic PSCs (presynaptic actions), in ~50% of GnRH neurons through D1-like and/or D2-like receptors (95). On the other hand, endogenous DA (from neurons in the RP3V and possibly elsewhere) increases firing in ~30% of GnRH neurons, whereas DA from RP3V neurons alone decreases firing in ~20% of GnRH neurons, also through D1-like and/or D2-like receptors (95). D1-like receptors are G_s_-coupled receptors that stimulate adenylyl cyclase (AC) to produce cyclic adenosine monophosphate (cAMP), which activates protein kinase A (PKA) and may in turn inhibit K^+^ channels, while D2-like receptors, including D2, D3, and D4 receptors that appear to be expressed in GnRH neurons (67, 70), are G_i_-coupled receptors that inhibit AC, which results in reduced cAMP levels, inhibition of PKA, and activation of K^+^ channels. Yet, it is unclear how activation of D1-like receptors decreases GnRH neuron firing, since they are coupled to G_s_, and since forskolin, which also stimulates AC and increases cAMP, increases GnRH neuron firing (96). DA, or tyrosine hydroxylase (TH), the rate-limiting enzyme in DA synthesis, is co-expressed in ~90% of RP3V KP neurons, and axon terminals of TH neurons appose GnRH neurons (97, 98), but mice in which TH has been knocked out exhibit normal puberty, LH levels, and fertility, suggesting that DA from RP3V KP neurons is not required for puberty or reproduction (99). However, other neurotransmitters may have compensated for the loss of TH (and likely absence of DA) in RP3V KP neurons in those mice.

#### Serotonin (5-HT)

GnRH neurons in the POA receive direct projections from 5-HT neurons in the raphe nuclei [RN (100)]. Exogenous 5-HT, or the selective serotonin reuptake inhibitor (SSRI) fluoxetine, which increases endogenous 5-HT levels, directly inhibits (via 5-HT1A receptors, which are coupled to G_i_) and then excites (via 5-HT2A receptors, which are coupled to G_q_) GnRH neurons (96). The inhibition depends on K^+^ channel activation and AC inhibition, whereas the excitation depends on protein kinase C (PKC), and the balance of 5-HT-evoked inhibition vs. excitation varies according to age, sex, and estrous cycle stage (96). However, it should be noted that preventing or blocking AC activity has also been shown to have no effect on GnRH neuron activity (101, 102). GnRH neurons appear to express 5-HT1A, 5-HT2A, 5-HT1A, 5-HT3A, and 5-HT4 5-HT receptor subunits (67, 69, 70).

#### Norepinephrine (NE)

Brainstem A2 (solitary tract nucleus; NTS) and A6 (locus coeruleus; LC) NE-containing neurons project to GnRH neurons in the POA (100), and dopamine-β-hydroxylase (the rate-limiting enzyme for NE synthesis)-immunoreactive terminals form synapses on GnRH neuron dendrites (103). NE suppresses GnRH neuron firing directly, and the suppression is mimicked by the α1-adrenergic agonist phenylephrine and β-adrenergic agonist isoproterenol, but not by the α2-adrenergic receptor agonist guanabenz, suggesting that NE activates α1- and β-adrenergic, but not α2-adrenergic receptors, in GnRH neurons (104). Additional evidence for the participation of both α1- and β-adrenergic receptors is that the α1-adrenergic antagonist prazosin reduces the hyperpolarizing action of NE significantly but not completely (104). However, it is unclear how α1- and β-adrenergic receptors mediate the suppression since α1- and β-adrenergic receptors are coupled to the stimulatory G proteins G_q_ and G_s_, respectively. GnRH neurons appear to express α1A-, α1B-, α2A-, α2B-, α2C-, and β1-adrenergic subunits (67, 69, 70).

#### Epinephrine (Epi)

Inhibition of central Epi synthesis blocks the GnRH/LH surge in E and P-treated ovariectomized rats (105); however, the effects of Epi, which like NE may bind to α1- and β-adrenergic receptors, on GnRH neuron activity or GnRH/LH secretion in mice have yet to be reported.

#### Histamine (H)

H increases [Ca^2+^]_i_ and GnRH secretion in immortalized mouse GnRH neurons via H1 receptors coupled to phosphoinositide hydrolysis (106, 107). Also, axons of ERα-expressing histaminergic neurons in the tuberomammillary nucleus (TMN) in rats and humans exhibit axo-dendritic and axo-somatic appositions onto GnRH neurons, and intracerebroventricular administration of the H1 receptor antagonist, mepyramine, blocks the GnRH/LH surge in ovariectomized E-treated rats, suggesting that E-receptive histaminergic neurons in the TMN contribute to the positive feedback effect of E in the induction of the GnRH/LH surge (108). Although mouse GnRH neurons appear to express H1 and H2 receptors (67, 70), which are coupled to G_q_ and G_s_, respectively, the effects of H on GnRH neuron activity and secretion in mice have yet to be reported.

### Purines

#### Adenosine triphosphate (ATP)

Extracellular ATP increases [Ca^2+^]_i_ and GnRH secretion via activation of P2X receptor channels, which are non-selective cation channels, followed by membrane depolarization and subsequent Ca^2+^ influx through L-type Ca^2+^ channels, in GnRH neurons cultured from the olfactory placode of monkey embryos (109). Mice express P2X2, P2X4, P2X5, P2X6, and P2X7 ATP receptor subunits in GnRH neurons (70, 110). While blockade of P2X receptor channels by the P2X receptor antagonist pyridoxal-phosphate-6-azophenyl-2′,4′-disulfonate (PPADS) has no effect on spontaneous [Ca^2+^]_i_ oscillations in GnRH neurons cultured from nasal pit explants of mouse embryos (27), there are no reports yet on the effects of extracellular ATP, or of P2X receptor antagonists, on GnRH neuron electrical activity, [Ca^2+^]_i_, or secretion in GnRH neurons of postnatal mice.

#### Adenosine (Ado)

GnRH neurons appear to express Ado receptors 2A and 2B (67, 70), which are coupled to G_s_. However, as with extracellular ATP, there are no reports yet on the effects of extracellular Ado on GnRH neuron electrical activity, [Ca^2+^]_i_, or secretion in mice.

### Cholinergics

#### Acetylcholine (ACh)

ACh exerts stimulatory and inhibitory effects on GnRH secretion in immortalized GnRH neurons, which express α7 nicotinic ACh receptors (AChRs) and M1, M2, and M4 muscarinic AChRs (111–113). Activation of α7 nicotinic ACh AChRs, which are non-selective cation channels, transiently increases basal GnRH secretion from immortalized GnRH neurons via depolarization followed by Ca^2+^ influx through voltage-gated Ca^2+^ channels (111, 112). It also inhibits prostaglandin E2 (PGE2)- and high K^+^-induced GnRH secretion (113), possibly due to Ca^2+^-dependent Ca^2+^ channel inactivation. Activation of M1 muscarinic AChRs, which couple to G_q_, with micromolar concentrations of ACh, increases basal GnRH secretion from immortalized GnRH neurons via IP_3_-mediated Ca^2+^ mobilization (111, 114), whereas activation of M2 and M4 muscarinic AChRs, which couple to G_i_, with nanomolar concentrations of ACh, inhibits basal GnRH secretion from immortalized GnRH neurons (111, 112). Most likely, ACh first increases [Ca^2+^]_i_ and GnRH secretion in immortalized GnRH neurons by activating α7 nicotinic AChRs, then decreases [Ca^2+^]_i_ and GnRH secretion by activating M2/M4 AChRs, and subsequently induces a return of [Ca^2+^]_i_ and GnRH secretion to basal levels by activating M1 muscarinic AChRs. Cholinergic axons appose, but do not make classical synapses onto, GnRH neurons in rats, suggesting a non-synaptic route of cholinergic communication to GnRH neurons (115). Mouse GnRH neurons appear to express β1, β2, β4, γ, and ε nicotinic AChRs as well as M1 and M4 muscarinic AChRs (67, 69, 70). However, the effects of ACh on GnRH neuron activity or secretion in mice, and whether cholinergic axons appose GnRH neurons in mice, have yet to be reported.

## Modulation of GnRH Neuron Activity and Secretion by Gasotransmitters

Gasotransmitters are defined here as small molecules of gas that are produced in the cytoplasm of neurons and immediately diffuse through the cell membrane into the extracellular fluid and into nearby neurons (e.g., GnRH neurons) to stimulate production of second messengers (such as cGMP) that can affect neuronal activity and neurotransmitter/neuropeptide release.

### Nitric Oxide (NO)

NO released from neuronal nitric oxide synthase (nNOS)-expressing neurons in the POA directly inhibits the firing of GnRH neurons in the POA by activating soluble guanylyl cyclase (sGC, the NO receptor) and a K^+^ conductance (16). The nNOS in the POA nNOS neurons (and thus, presumably, NO release from those neurons) is required for E-mediated feedback inhibition of pulsatile GnRH/LH release, as well as for the proestrus GnRH/LH surge, gonadal development, and fertility (28, 116). POA nNOS neurons are apposed by KP-immunoreactive fibers, express the kisspeptin receptor, G protein-coupled receptor 54 (GPR54), and exhibit increased nNOS phosphorylation/activation in response to both E and KP (28). However, the POA nNOS neurons, unlike GnRH neurons, appear not to be the key site of KP-GPR54 signaling for fertility, since GnRH neuron-specific deletion of GPR54 prevents gonadal/pubertal development and results in infertility while GnRH neuron-specific rescue of GPR54 in global GPR54 knockout mice results in normal pubertal development and fertility (3). In addition to nNOS expression in POA neurons, GnRH neurons themselves express nNOS and are capable of generating NO: the retrograde messenger acts on presynaptic GABA and Glu boutons that contain sGC and, as mentioned above, increases GABAergic and glutamatergic transmission to GnRH neurons in response to E and may contribute to the proestrous GnRH/LH surge (86, 117).

### Carbon Monoxide (CO)

Although the heme oxygenase 1 (HO-1) activator/CO donor hematin was reported to have no effect on *in vivo* release of GnRH from rat hypothalamus (118), the HO-1 donor hemin was reported to increase GnRH release from immortalized GnRH neurons (119). However, there are no reports yet on the effect of CO on GnRH neuron activity or secretion in mice.

## Modulation of GnRH Neuron Activity and Secretion by Gliotransmitters

Gliotransmitters are defined here as endogenous chemicals released from glial cells (primarily astrocytes), through plasma membrane channels, plasma membrane transporters, or Ca^2+^-dependent exocytosis onto neurons (e.g., GnRH neurons), where they bind to and activate specific receptors linked to ion channels or second messenger pathways. Similar to neurotransmission, gliotransmission results in a change or modulation of the activity (electrical activity and/or [Ca^2+^]_i_) and output (neurotransmitter or hormone secretion) of the neurons.

### Prostaglandin E2 (PGE2)

PGE2 released from astrocytes in response to neuregulin-erbB signaling acts directly on GnRH neuron cell bodies in the POA, which morphologically interact with astrocytes, via the EP2 class of PGE2 receptors and PKA signaling to increase GnRH neuron firing and GnRH secretion (17, 120). It may mediate the stimulatory effects of Glu, oxytocin and/or blood-borne factors on GnRH neurons (17, 120) and is necessary for the normal timing of puberty and for adult reproductive function (121, 122).

### Other Gliotransmitters?

Some of the non-peptide neurotransmitters, such as GABA, Glu, and ATP, may also function as gliotransmitters [reviewed by (123–125)]. Whether they do so to modulate GnRH neuron activity and secretion remains to be explored. In addition, microglia-derived cytokines, including interleukin-1 (IL-1), interleukin-10 (IL-10), and interleukin-18 (IL-18), which are proteins, as well as microglia-derived prostaglandins, may function as gliotransmitters and together with their receptors expressed in GnRH neurons appear to be important in modulating GnRH neuron activity, especially under inflammatory conditions (126–132).

## Concluding Remarks

**Figure 1:**
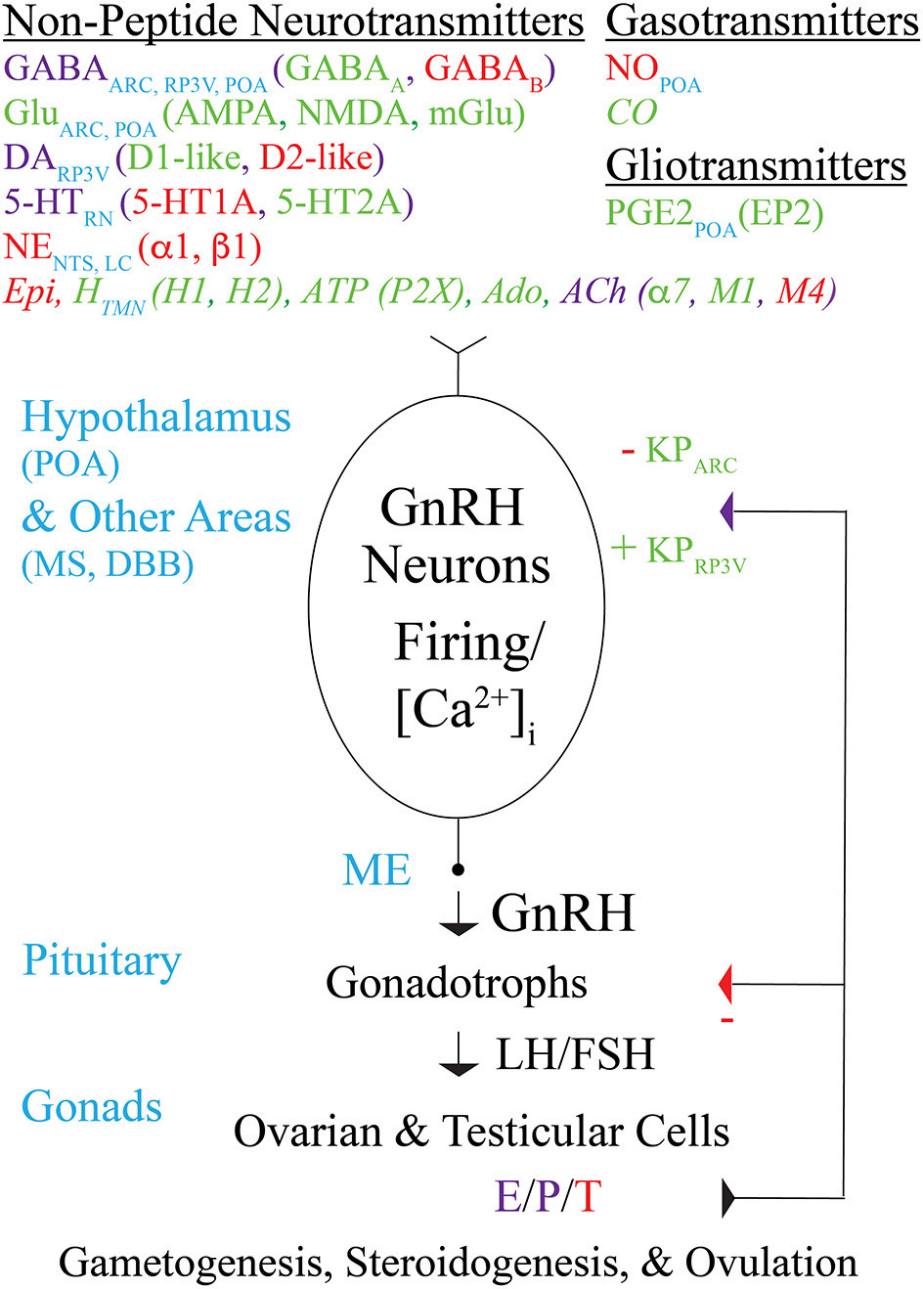
Modulation by non-peptide neurotransmitters, gasotransmitters, and gliotransmitters of GnRH neuron activity and GnRH secretion controlling fertility in mice. Schematic diagram showing non-peptide neurotransmitters (and their receptors), gasotransmitters, and gliotransmitters that act directly on the cell bodies, dendrites, axons, and/or “dendrons” of GnRH neurons to modulate their action potential firing, [Ca^2+^]_i_, and GnRH secretion. Transmitters that excite, inhibit, or both excite and inhibit GnRH neurons are indicated in green, red, or purple, respectively, and are listed along with the brain areas in which they are produced. Transmitters that modulate GnRH neuron activity and/or secretion in immortalized GnRH neurons, rat GnRH neurons, or monkey GnRH neurons but have not yet been reported to modulate GnRH neuron activity or secretion in mice are indicated in italics. GnRH secreted from GnRH neuron axon terminals in the ME into the hypothalamo-hypophyseal circulation binds to GnRH receptors on pituitary gonadotrophs to stimulate the synthesis and secretion of LH and FSH into the general circulation. LH and FSH, which are required for gametogenesis and ovulation, bind to receptors in the gonads to stimulate the synthesis and secretion of E, P, and T, which in turn exert negative or positive feedback effects on GnRH neurons (via KP neurons) and gonadotrophs depending on the sex and estrous cycle stage of the animal. Abbreviations are explained at their first occurrence in the main text.

As described above, and indicated in Figure 1, various non-peptide neurotransmitters, gasotransmitters, and gliotransmitters modulate GnRH neuron activity and GnRH secretion controlling fertility in mice. Modulation by some transmitters has been demonstrated in immortalized mouse GnRH neurons, rats, or other mammalian species but not yet in mice. Further research is needed to address whether those transmitters modulate GnRH neuron activity or GnRH/LH secretion in mice. Research is also needed to determine which presynaptic neurons and glial cells release which transmitters onto GnRH neurons, and under which physiological conditions, as well as the roles of selected transmitter receptors of interest in GnRH neurons on GnRH neuron activity, GnRH/LH secretion, and fertility. Approaches such as rabies viral monosynaptic tracing [to identify the cells that make direct synaptic connections onto GnRH neurons (133)] and inducible GnRH neuron-specific deletion of selected transmitter receptor subunits [to avoid developmental compensation by other subunits or transmitters (134)], should help in this regard. *In vivo or ex vivo* recording, imaging, or photometry of GnRH neuron electrical activity or Ca^2+^ dynamics (5, 19, 25, 135), in combination with optogenetic or chemogenetic stimulation or inhibition of presynaptic cells [to evoke or inhibit the release of transmitters from presynaptic cells (4, 26, 135–138)], along with new or improved methods for measuring GnRH/LH secretion, and monitoring of fertility (22, 137, 139, 140), should also help. Finally, to better understand how endocrine, metabolic, and environmental signals integrate into the HPG axis to control fertility, in addition to elucidating the direct modulation of GnRH neuron activity and secretion by transmitters, it will be important to further delineate the upstream networks that connect to KP neurons, other neurons, and glial cells that release the transmitters that affect GnRH neuron [see for example (141)].

## Author Contributions

The author confirms being the sole contributor of this work and has approved it for publication.

### Conflict of Interest Statement

The author declares that the research was conducted in the absence of any commercial or financial relationships that could be construed as a potential conflict of interest.
